# Optical properties of ZnO/BaCO_3_ nanocomposites in UV and visible regions

**DOI:** 10.1186/1556-276X-9-399

**Published:** 2014-08-18

**Authors:** Ali Khorsand Zak, Abdul Manaf Hashim, Majid Darroudi

**Affiliations:** 1 Malaysia-Japan International Institute of Technology (MJIIT), University Teknologi Malaysia (UTM), Jalan Semarak, Kuala Lumpur 54100, Malaysia; 2 Nanotechnology Laboratory, Esfarayen University of Technology, Esfarayen 96619-98195, North Khorasan, Iran; 3 Department of Modern Sciences and Technologies, School of Medicine, Mashhad University of Medical Sciences, Mashhad 3316-913791, Iran

**Keywords:** Optical, Composite materials, Ceramic materials

## Abstract

Pure zinc oxide and zinc oxide/barium carbonate nanoparticles (ZnO-NPs and ZB-NPs) were synthesized by the sol–gel method. The prepared powders were characterized by X-ray diffraction (XRD), ultraviolet–visible (UV–Vis), Auger spectroscopy, and transmission electron microscopy (TEM). The XRD result showed that the ZnO and BaCO_3_ nanocrystals grow independently. The Auger spectroscopy proved the existence of carbon in the composites besides the Zn, Ba, and O elements. The UV–Vis spectroscopy results showed that the absorption edge of ZnO nanoparticles is redshifted by adding barium carbonate. In addition, the optical parameters including the refractive index and permittivity of the prepared samples were calculated using the UV–Vis spectra.

**PACS:**

81.05.Dz; 78.40.Tv; 42.70.-a.

## Background

Nanotechnology has the potential to create many new devices with a wide range of applications in the fields of medicine [1], electronics [2], and energy production [3]. The increased surface area-to-volume ratios and quantum size effects are the properties that make these materials potential candidates for device applications. These properties can control optical properties such as absorption, fluorescence, and light scattering. Zinc oxide (ZnO) is one of the famous metal oxide semiconductors with a wide bandgap (3.36 eV) and large excitation binding energy. These special characteristics make it suitable to use in many applications, such as cancer treatments [4], optical coating [5], solar cells [3], and gas sensors [6]. In fact, doping, morphology, and crystallite size play an important role on the optical and electrical properties of ZnO nanostructures, which can be controlled by methods of the nanostructure growth. Therefore, many methods have been created to prepare ZnO nanostructures including sol–gel [7], precipitation [8], combustion [9], microwave [10], solvothermal [11], spray pyrolysis [12], hydrothermal [13,14], ultrasonic [15], and chemical vapor deposition (CVD) [16,17]. As mentioned above, the doping of ZnO with selective elements offers an effective method to enhance and control its electrical and optical properties. The effects of several elements on the optical and electrical properties of ZnO material have been investigated. For example, Au^2+^[18], Ce^3+^[19], Eu^3+^[20], In^3+^[21], and Mg^2+^[22,23] have been used in order to control the optical properties; Mn^2+^[24], Cr^2+^[25], Co^2+^, Ni^2+^, Fe^3+^, Cu^2+^, and V^5+^[26] have been used to enhance the magnetic properties; and Li^1+^ and Na^1+^[27] have been used to obtain a p-type form of ZnO.

In the present research, a modified sol–gel route was used to prepare ZnO/BaCO_3_ nanoparticles (*x* = 0, ZnO-NPs; *x* = 0.1, ZB10-NPs; *x* = 0.2, ZB20-NPs) using gelatin as a polymerization agent. The gelatin was used as a terminator for growing the ZnO/BaCO_3_-NPs because it expands during the calcination process and the particles cannot come together easily. The crystallite size and crystallinity of the resulting ZnO/BaCO_3_-NPs were investigated.

## Methods

In order to synthesize zinc oxide/barium carbonate nanoparticles (ZB-NPs), analytical-grade zinc nitrate hexahydrate (Zn(NO_3_)_2_ · 6H_2_O, Sigma-Aldrich, St. Louis, MO, USA), barium nitrate (Ba(NO_3_)_2_, Sigma-Aldrich), and gelatin [(NHCOCH-R_1_)_*n*_, R_1_ = amino acid, type b, Sigma-Aldrich] were used as starting materials and distilled water as solvent. To prepare 10 g of the final product (ZB-NPs), the appropriate amounts of zinc and barium nitrate were dissolved in 50 ml of distilled water. The amounts of the precursor materials were calculated according to the (1 - *x*)ZnO/(*x*)BaCO_3_ formula, where *x* = 0, 0.1, and 0.2. On the other hand, 8 g of gelatin was dissolved in 300 ml of distilled water, and the solution was stirred at 60°C to obtain a clear gelatin solution. Finally, the Zn^2+^/Ba^2+^ solution was added to the gelatin solution. The container was then moved into an oilbath; meanwhile, the temperature of the oilbath was kept at 80°C while being continuously stirred to achieve a viscose, clear, and honey-like gel. For the calcination process, the gel was slightly rubbed on the inner walls of a crucible and then placed into the furnace. The temperature of the furnace was fixed at 650°C for 2 h, with a heating rate of 2°C/min.

The phase evolutions and structure of the prepared pure zinc oxide nanoparticles (ZnO-NPs) and ZB-NPs were investigated by X-ray diffraction (XRD; Philips X'pert, Cu K_α_, Philips, Amsterdam, the Netherlands). The transmission electron microscopy (TEM) observations were carried out on a Hitachi H-7100 electron microscope (Hitachi Ltd., Chiyoda-ku, Japan) to examine the shape and particle size of the nanoparticles and field emission Auger electron spectroscopy (AES; JAMP-9500 F, JEOL Ltd., Akishima-shi, Japan) for elemental analysis. The ultraviolet–visible (UV–Vis) spectra were recorded by a PerkinElmer Lambda 25 UV–Vis spectrophotometer (PerkinElmer, Waltham, MA, USA).

## Results and discussion

### XRD analysis

**Figure 1:**
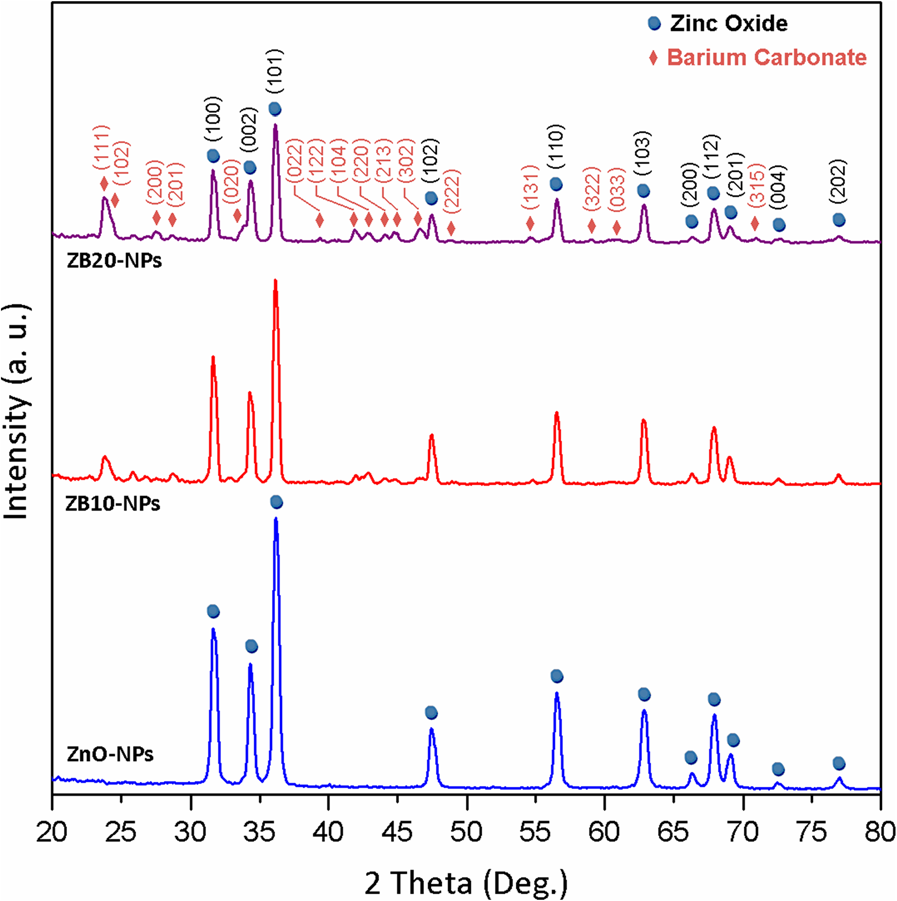
XRD patterns of the synthesized ZnO and ZB nanoparticles.

**Figure 2:**
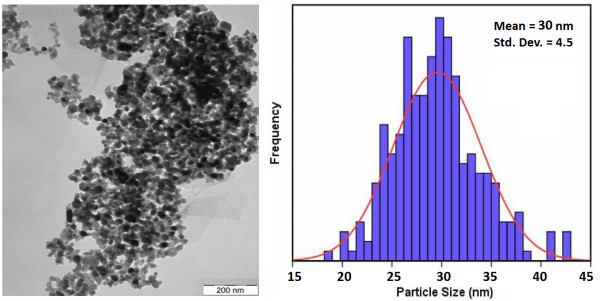
Typical TEM image of the ZB20 nanoparticles and the corresponding size distribution histogram.

XRD patterns of the synthesized pure ZnO-NPs and ZB-NPs are shown in Figure 1. It is observed that the orthorhombic BaCO_3_ nanostructures (PDF card no: 00-041-0373) have been grown besides the hexagonal ZnO nanocrystals (ref. code no: 00-001-1136) as indexed in the pattern. It is indicated that the ZnO and BaCO_3_ nanocrystals have been grown independently. No other diffraction peak related to the other compounds or impurities was detected. The crystallite sizes of the ZnO/BaCO_3_ nanoparticles were calculated using the Scherrer equation and obtained to be 17 ± 2, 18 ± 2, and 21 ± 2 nm, respectively. The calculations were applied on the ZB-NPs XRD pattern using parameters related to the (101) (for ZnO) diffraction peaks. A typical TEM image of ZB20-NPs is presented in Figure 2. The average particle size of the ZB20-NPs was obtained to be about 30 nm. It can be seen that the average value of the measured particle sizes is in good agreement with the calculated crystallite sizes as expected.

### UV–Vis diffuse reflectance spectra and bandgap

**Figure 3:**
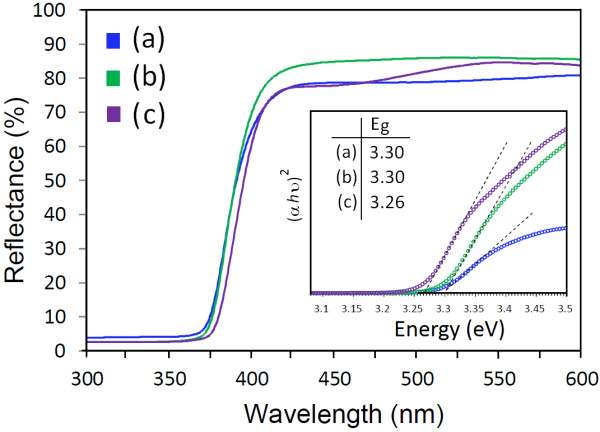
**The reflectance spectra of the synthesized (a) ZnO, (b) ZB10, and (c) ZB20 nanoparticles.** The inset shows the obtained optical bandgap using the Kubelka-Munk method.

UV–Vis reflectance spectra of the pure ZnO-NPs and ZB-NPs prepared at a calcination temperature of 650°C are shown in Figure 3. The relevant increase in the reflectance at wavelengths bigger than 375 nm can be related to the direct bandgap of ZnO due to the transition of an electron from the valence band to the conduction band (O_2p_ → Zn_3d_) [28]. An obvious redshift in the reflectance edge was observed for ZB-NPs compared to the pure ZnO. As obtained in the ‘XRD analysis’ section, the crystallite size of the ZnO nanoparticles is increased by adding BaCO_3_; therefore, this redshift can be related to the quantum confinement effect or quantum size effects. This might be due to changes in their morphologies, crystallite size, and surface microstructures of the ZnO nanocrystals besides the BaCO_3_ nanocrystals. The result of the UV–Vis spectroscopy can be used for calculating the optical bandgap of the materials. Using the Kubelka-Munk model is a way to calculate the optical bandgap, while the direct bandgap energies can be estimated from a plot of (*αhν*)^2^ versus the photon energy (*hν*) [22]. This method has been obtained from the Tauc relation, which is given by [29]

(1) α=Ahvhv-Eg1m

where *A* is a constant and *m* = 2 when the bandgap of the material is direct. Also, the absorption coefficient can be obtained from [30]

(2) α=1-R′22R′R′=R100

where *R* is the reflectance.

The derivative method has been found as an easy and accurate method to calculate the optical bandgap compared to the Kubelka-Munk method. In this method, the direct bandgap can be estimated from the maximum of the first derivative of the absorbance data plotted versus energy or from the intersection of the second derivative with energy axis.

**Figure 4:**
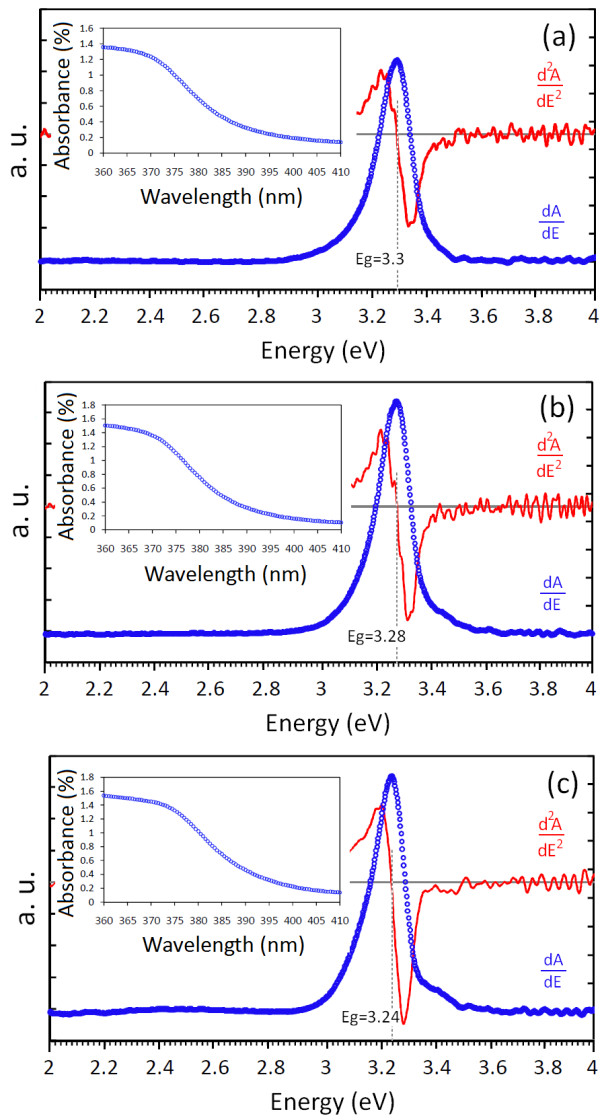
**Optical bandgap value of the synthesized (a) ZnO, (b) ZB10, and (c) ZB20 nanoparticles.** The absorbance is shown in the inset.

The energy bandgap of the synthesized samples at 650°C was estimated from the methods mentioned above. The optical bandgaps of the ZB*x*-NPs (*x* = 0, 10, and 20) calculated by the Kubelka-Munk method were obtained to be 3.30, 3.30, and 3.26 eV, respectively, as shown in the inset of Figure 3. The absorbance spectra and their corresponding first and second derivatives are drawn in Figure 4a,b,c, and the bandgaps of 3.30, 3.28, and 3.24 were estimated for ZnO, ZB10, and ZB20 nanoparticles, respectively. It can be seen that the bandgap of the ZnO nanoparticles decreased by adding barium. As mentioned earlier, the crystallite size of the prepared nanoparticles increased by adding barium, resulting to redshifting of the absorption edge due to the quantum confinement and size effects. The bandgap is estimated from the absorption spectrum; therefore, the value of the obtained bandgap decreased for the barium-added samples. Considering the results obtained from the methods, it can be concluded that there is a better agreement between the derivative method with the observed blueshift in reflectance spectra and the Kubelka-Munk method due to the less approximations of the derivative method.

### Method of optical constant calculations

In the complex refractive index, *N = n - ik*, *n* is the refractive index and *k* is the extinction coefficient. The extinction coefficient is related to the absorption coefficient by *k = λα/*4*π*. According to the Fresnel formula, the reflectance as a function of the refractive index *n* and the absorption index *k* is given as [31]

(3) $$R^{\prime }=\frac{{\left(n-1\right)}^{2}+k^{2}}{{\left(n+1\right)}^{2}+k^{2}}$$

As mentioned above, the extinction coefficient is obtained using *k = λα/*4*π*, where the absorption coefficient is calculated from Equation 3. Therefore, by calculating *α* and then *k*, the refractive index can be obtained from

(4) $$n=\left(\frac{1+R^{\prime }}{1-R^{\prime }}\right)+\sqrt{\frac{4R^{\prime }}{{\left(1-R^{\prime }\right)}^{2}}-k^{2}}$$

According to the obtained results for *n* and *k*, the real and imaginary parts of the dielectric function can be calculated by the following equations [32]:

(5) $$\begin{array}{l} \tilde{\varepsilon }=\varepsilon^{\prime }+i\varepsilon^{\prime \prime }\\ \varepsilon^{\prime }=n^{2}-k^{2}\\ \varepsilon^{\prime \prime }=2\text{nk} \end{array}$$

**Figure 5:**
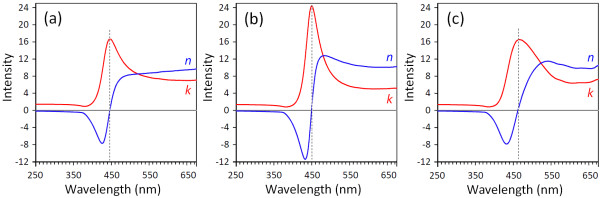
**The behavior of the refractive indexes and extinction coefficients calculated near the absorption edge. (a)** ZnO, **(b)** ZB10, and **(c)** ZB20 nanoparticles.

**Figure 6:**
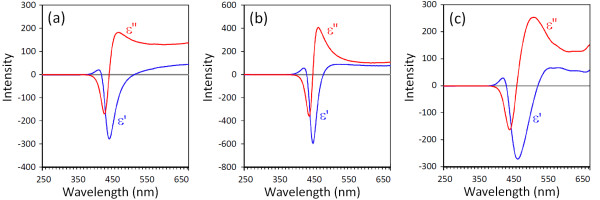
**The behavior of the real and imaginary parts of permittivity calculated near the absorption edge. (a)** ZnO, **(b)** ZB10, and **(c)** ZB20 nanoparticles.

The obtained results for the optical properties are presented in Figures 5 and 6.

### Auger spectroscopy of ZnO/BaCO_3_ nanocomposites

**Figure 7:**
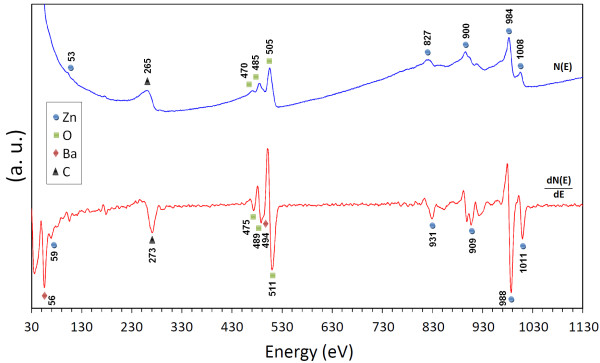
The Auger spectrum of the synthesized ZB20 nanoparticles.

Auger spectroscopy is a helpful method to be used for element detection of compounds. Figure 7 shows the high-resolution N(E) (blue line) and related derivative (red line) AES of the ZB-NPs calcined at 650°C. The Auger spectra of barium, oxygen, carbon, and zinc were indexed in the Auger spectrum. The derivative AES spectrum of barium indicates peaks at 56 and 494 eV, corresponding to the MVV and KLL derivative Auger electron emission from barium. In the middle part of the figure, which relates to oxygen, the Auger spectrum indicates peaks at 470, 485, and 505 eV. These peaks can be attributed to the KLL Auger electron emission of oxygen [33]. Finally, the spectra of zinc are shown in Figure 7. The LMM Auger electron emission peaks of zinc are detected at 827, 900, 984, and 1,008 eV and the MVV at 53 eV [30]. No further Auger electron emissions related to the other elements are observed in this energy region.

## Conclusions

ZnO and ZnO/BaCO_3_ nanoparticles were synthesized by the sol–gel method. XRD was used to study the crystallite sizes and structures. The crystallite sizes of the prepared BaCO_3_ and ZnO nanoparticles were obtained to be 12 ± 2 and 21 ± 2 nm, respectively, for ZB20-NPs. The average particle size of the prepared ZB20-NPs was obtained to be 30 nm, which supports the XRD results. The optical properties of the prepared samples were studied using UV–Vis spectroscopy. The analyzed results showed that the resonance frequency of the refractive index and permittivity is redshifted by BaCO_3_ concentration increases. The bandgaps of the pure ZnO, ZB10, and ZB20 nanoparticles were estimated to be 3.3, 3.28, and 3.24, respectively.

## Competing interests

The authors declare that they do not have competing interests.

## Authors’ contributions

AKZ carried out the sample preparation, XRD, and UV section. MD carried out the TEM imaging and Auger spectroscopy part. AMH was the project leader and contributed in analyzing the data. All authors read and approved the final manuscript.
